# Epiphitic Microbiome of Alvarinho Wine Grapes from Different Geographic Regions in Portugal

**DOI:** 10.3390/biology12020146

**Published:** 2023-01-18

**Authors:** Paulo Fernandes, Isabel Maria Afonso, Jéssica Pereira, Rui Rocha, Ana Sofia Rodrigues

**Affiliations:** 1 CISAS, Escola Superior de Tecnologia e Gestão, Instituto Politécnico de Viana do Castelo, Rua Escola Industrial e Comercial de Nun’Álvares, 4900-347 Viana do Castelo, Portugal; 2 CISAS, Escola Superior Agrária, Instituto Politécnico de Viana do Castelo, Rua Escola Industrial e Comercial de Nun’Álvares, 4900-347 Viana do Castelo, Portugal; 3 Escola Superior Agrária, Instituto Politécnico de Viana do Castelo, Rua Escola Industrial e Comercial de Nun’Álvares, 4900-347 Viana do Castelo, Portugal

**Keywords:** grape microbiome, *Alvarinho*, metagenomic, terroir

## Abstract

**Simple Summary:**

Wine, the product of biological activity on grape must, is much more than a simple product of fermentation. In fact, its relevance is manifested through cultural and social impacts, and it is, in many cases, a factor of economic development and fundamental regional/local identity. *Alvarinho* monovarietal wine is an example of this, as it is originally from the sub-region of Monção and Melgaço in the North of Portugal. The characteristics of a wine are highly influenced by several factors, including the microbial flora present on the surface of the grapes. This microbiota will contribute to the *terroir* associated with a given region in the production of wine, allowing the creation of wines with specific characteristics and greater typicality. The microbiome present on the surface of *Alvarinho* grapes was analyzed by High-Throughput Sequencing, and the results allowed for the determination of a core microbiome associated with grapes from any of the analyzed regions. The results made it possible also to verify that the α-diversity (for fungi) of the microbial population of the grapes from the region where the *Alvarinho* variety is native is different, with a yeast of the genus *Metschnikowia*, which is described as having enological potential in the creation of fresh and fruity wines, ubiquitously present in all plots of Monção and Melgaço.

**Abstract:**

Geographic location and, particularly, soil and climate exert influence on the typicality of a wine from a specific region, which is often justified by the *terroir*, and these factors also influence the epiphytic flora associated with the surface of the grape berries. In the present study, the microbiome associated with the surface of berries obtained from ten vineyards of the *Alvarinho* variety located in different geographical locations in mainland Portugal was determined and analyzed. The removal of microbial flora from the surface of the berries was carried out by washing and sonication, after which the extraction and purification of the respective DNA was carried out. High-throughput short amplicon sequencing of the fungal ITS region and the bacterial 16S region was performed, allowing for the determination of the microbial consortium associated with *Alvarinho* wine grapes. Analysis of α-diversity demonstrated that parcels from the Monção and Melgaço sub-region present a significantly (*p* < 0.05) lower fungal diversity and species richness when compared to the plots analyzed from other regions/sub-regions. The ubiquitous presence of *Metschnikowia* spp., a yeast with enologic potential interest in all parcels from Monção and Melgaço, was also observed.

## 1. Introduction

The surface of fruits, independently of the species and variety, is commonly colonized by microbial communities. In the case of grape berries that are used to make wine, these microbial communities, besides their influence on fruit health, may also have a crucial role in, or at least a significative influence on, the development of the organoleptic characteristics of the wine. This effect results from the unavoidable and sometimes even desired presence of the microbial communities in the must and during the wine production process. Given the potential origin of these microorganisms and that a large number of factors may influence their growth and development, it is understandable that soil richness and composition, temperature, humidity, UV radiation, chemicals/pollutants and all edapho-climatic factors as well as plant species [1,2] and ripening stage may have a great influence on the structure of the microbial communities in grape berries [1,3]. Microbial distribution patterns can, therefore, be geographically related [4,5] and contribute to the character of a certain type of a wine in a region [6]. These distribution patterns thus contribute to the so-called *terroir*, the “delimited area where the natural environment, the physical and chemical features of the soil, and climate conditions allow the achievement of specific grape characteristics, so that the obtained wine can be identified by means of the unique traits of its territoriality” [4].

The sub-region of Monção and Melgaço, which developed around the south bank of the River Minho, right up in the north of the Vinho Verde region in the north of mainland Portugal and near the Atlantic Ocean, has very particular climate conditions, with hot summers and abundant rainfall in winter. The specific characteristics of its geographical location and, in particular, the proximity to the Gerês Mountains make this region significantly different from the others within the Vinho Verde region, presenting unique microclimates that naturally influence the wines produced there [7]. *Alvarinho* wines are its main wine reference, and Monção and Melgaço is where the *Alvarinho* grape variety has its origin and where it began its evolution. It was one of the first Portuguese grape varieties to be bottled as a single variety, and its full-bodied, subtly fragrant white wines are easy to recognize, as they have complex but delicate aromas [8]. The objectives of the present study were: (i) to provide an exhaustive characterization of both the fungal and bacterial communities associated with the surface of *Alvarinho* grape berries during the harvest period; (ii) to compare the microbial communities associated with the grape berries from four different enological regions of Portugal, namely Vinhos Verdes, Dão, Alentejo and Trás-os-Montes; and (iii) to identify potential microbial signatures that may contribute to the specific *terroir* of wines produced in the Monção and Melgaço sub-region.

## 2. Materials and Methods

### 2.1. Collection and Transport of Grape Samples

A total of ten parcels in seven sub-regions from four distinct wine regions across Portugal (Figure S1 and Table 1) were sampled. In each parcel, 6 sub-samples of grapes were collected shortly before grape harvesting, within a radius of no more than 5 m around the sampling point. A composite sample (after mixing all the sub-samples) of about 3 kg of visually sound bunches was obtained and kept at 4 °C in a sterile plastic bag until processed (within 12 to 24 h after collection). All the vineyards sampled are run according to the principles of integrated production and subjected to similar agricultural and agronomic practices.

### 2.2. Extraction of the Microbial Flora Associated with the Grapes Surface

From each composite sample, 40 sound and intact grape berries with pedicel were cut with a sterile blade and washed with 80 mL of 0.1% (*w*/*v*) Buffered Peptone Water (Merck, Darmstadt, Germany) with 0.01 % (*w*/*v*) Tween 80 (PanReac Applichem, Barcelona, Spain) for 30 min at room temperature in an orbital shaker (Certomat B. Braun, Melsungen, Germany) at 150 rpm followed by 30 s in an ultrasound bath (Soltec EP, Sonica, Milan, Italy) at 40 kHz. A total of ten samples were therefore extracted.

### 2.3. Extraction and Purification of Microbial DNA

The suspension obtained in Section 2.2 (20 mL) was centrifuged at 10,000× *g* for 5 min, and the pellet was subjected to DNA extraction using ZymoBIOMICSTM DNA Miniprep Kit (ZymoResearch, Irvine, CA, USA), as per the manufacturer’s instructions. Each sample was homogenized for 6 min at maximum speed, cleaned and concentrated using a DNA Clean and ConcentratorTM Kit (ZymoResearch, Irvine, CA, USA), as per the manufacturer’s instructions. The purified DNA from three independent extractions of each sample was pooled and stored at −20 °C until further analysis. Quantification of the extracted and purified DNA was performed by fluorimetry using Qubit 3.0 (Thermo Fisher Scientific, Waltham, MA, USA). PCR amplification with the extracted DNA was performed using primers targeting from V3 to V4 regions of the 16S rRNA gene for bacterial identification and the ITS2 region for fungi identification. The primers used were 341F (5′-CCTACGGGNGGCWGCAG-3′) and 805R (5′-GACTACHVGGGTATCTAATCC-3′) for bacterial DNA amplification, and the primers used were UTS3 (5′-GCATCGATGAAGAACGCAGC-3′) and ITS4 (5′-TCCGCTTATTGATATGC-3′) for fungal DNA amplification.

### 2.4. Bioinformatics

Raw reads were extracted from the Illumina MiSeq^®^ System in FASTQ format and quality filtered with Trimmomatic software [9] for removal of sequencing adapters, quality check and filtering of low quality (<Q25) reads. The resulting reads were then imported into the EzBioCloud platform (https://www.ezbiocloud.net, accessed on 24 June 2021) to filter chimeric-reads and generate a list of Operational Taxonomic Units (OTU) by using a >97% similarity threshold and, for taxonomic assignment, using internally curated databases [10]. For the calculation of microbial diversity estimators, the quality filtered read files were imported and analyzed using QIIME software version 2020.8 [11]. Forward and reverse reads were truncated at position 245 and merged by overlapping paired end reads using the *q2-dada2* plugin and the *denoise-paired* pipeline [12]. Chimeric merged reads were detected and removed using the *q2-vsearch* plugin and the *uchime-ref* pipeline [13] against Greengenes (version 13_8) [14] and UNITE (version 8.2, dynamic fungi release) [15] databases for bacterial and fungi files, respectively. The *q2–phylogeny* plugin and *align–to–tree–mafft–fasttree* pipeline [16] were used to create a phylogenetic tree. These were subsequently used in *q2–diversity* plugin and *core–metrics* pipeline to: (a) estimate α–diversity metrics, including Faith phylogenetic diversity [17], evenness, observed features and Shannon [18]; (b) estimate β–diversity metrics, including Bray–Curtis dissimilarity [19], Jaccard distance [20] and unweighted and weighted UniFrac [21]; and finally, (c) generate principal coordinates analysis (PCoA) plots. For these estimations, the sampling depth was set as the highest possible while retaining all of the samples. Additionally, the *q2–diversity* plugin and the *alpha–group–significance* and *beta–group–significance* pipelines were used to explore and disclose α and β–diversity differences among parcels, using Kruskal–Wallis [22] and permutational multivariate analysis of variance tests [23], respectively. Differences with *p* < 0.05 were considered significant. In order to identify differentially abundant *taxa* between and within parcels with possible biological significance, a Linear Discriminant Analysis (LDA) of Effect Size (LEfSe) was executed on a Galaxy computational tool (http://huttenhower.sph.harvard.edu/galaxy/, accessed on 21 June 2022). The analysis parameters were set as follows: 0.1 alpha value for the factorial Kruskal–Wallis test among classes; 0.05 alpha value for the pairwise Wilcoxon test between subclasses; and “one against all” strategy for multi-class analysis and a pairwise comparison only among subclasses with the same name.

### 2.5. Nucleotide Sequences Accession Number

Raw reads were deposited in the SRA database under BioProject PRJNA885087.

## 3. Results and Discussion

### 3.1. Bacterial Composition of the Alvarinho Grape Berries Surface

The surface of grapes is a relevant source of microorganisms present in wine must; hence, it influences the fermentation and the physicochemical and organoleptic characteristics of the wine produced. The presence of bacteria on the surface of grape berries largely depends on the health of the grapes and can significantly influence the quality of the wines obtained, despite the fact that most of the bacteria often found in grape samples, such as *Enterobacter* spp., *Enterococcus* spp., *Bacillus* spp., *Burkholderia* spp., *Serratia* spp. and *Staphylococcus* spp., do not have the capacity to grow in wines [5]. Mezzasalma and colleagues [24] have found a core composition of bacterial flora comprising *Enterobacteriales*, *Pseudomonadales*, *Bacillales*, and *Rhodospirillales* in all the sampled parcels of the Cannonau variety in Sardinia, Italy. The bacterial community at the surface of grape berries also seems to be highly dependent on the ripening stage, as it is particularly abundant initially on *Pseudomonas* but increasingly dominated by Gram-positive bacteria as ripening succeeds [25].

In the present study, we have obtained a total of 300,159 16S rRNA gene sequences from grape berries from ten different locations across Portugal, corresponding to 11 bacterial phyla, 84 families, 149 genera and 246 species associated with the surface of grape berries. The relatively high number of species found must be viewed with due caution, particularly because Illumina sequencing is prone to errors, as any other sequencing method, and the assignment of sequences to a specific OTU group can also be influenced by the database and the threshold used (97% identity in our case). At the genus level, the results showed that an average of about 30 different genera were identified for samples from parcels A to J, with a maximum of 81 genera in the parcel from the Lima sub-region and a minimum of 13 in the parcel from the Basto sub-region. In general, the rarefaction curves showed a stabilizing tendency, indicating sufficient sampling of microbial communities. At the phylum level, *Proteobacteria* were by far the most prevalent bacteria, as they were present in all the sampled parcels and accounted for slightly over 93% of the total reads. Firmicutes were also present in all analyzed parcels, though at considerably much lower proportions when compared to *Proteobacteria*. Only one other phyla, *Actinobacteria* (6.4%), was found to represent ≥ 1% of total reads but only in samples obtained in the parcel from the Lima sub-region. 

**Figure 1:**
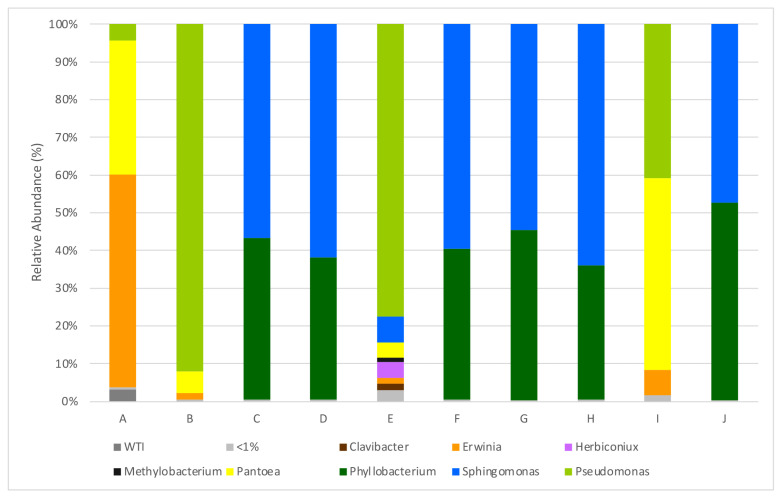
Relative abundances (%) of dominant sequences (>1%) assigned to genus level that were identified in samples based on partial sequence analysis of the V3–V4 regions of the 16S rRNA gene. WTI refers to the percentage of sequences without taxonomic attribution to the specified taxonomic level. A–J refers to the origin of the sequenced sample, as described in Table 1.

A total of nine genera with abundance greater than 1% of total reads were identified, and five of these genera showed abundance higher than 5%, namely *Sphingomonas* (in seven parcels), *Phyllobacterium* (in six parcels), *Pantoea* (in three parcels), *Pseudomonas* (in three parcels) and *Erwinia* (in two parcels), all of which are within the *Proteobacteria* phylum. As it can be observed in the histograms (Figure 1), there are significant differences among samples obtained from different parcels. In six of the analyzed plots, *Sphingomonas* and *Phillobacterium* are the dominant families; whereas in the other four, a greater abundance of *Pseudomonas* and *Erwinia* is observed. *Erwinia billingiae* is present in all parcels but significatively in parcel A from the Monção and Melgaço sub-region, accounting for 55.8% of all read counts. Some *Erwinia* species—such as *Erwinia amylovora*, the causative agent of fire blight, and *E. pyrifoliae*—are described as pathogens, but *Erwinia billingiae* is usually considered to be epiphytic [26,27]. *Pseudomonas lutea* is also present in high amounts but in parcel I from the Dão region. 

### 3.2. Fungal Composition of the Alvarinho Grape Berries Surface

Naturally, the environment in which the vine is inserted, namely the soil, leaves and bark, which are usually rich in basidiomycetous oxidative yeasts, influences the fungal composition of the surface of the grape berries. However, healthy grape berries are nutritionally poor environments, thus limiting the diversity of species capable of establishing themselves in this ecosystem and favoring oligotrophic, oxidative basidiomycetous yeasts [5]. However, as we can observe in the published literature, a significant variation of the microbial flora associated with the surface of grape berries is found, which is easily justified by all the factors affecting the growth and colonization of these biological agents [5,28].

**Figure 2:**
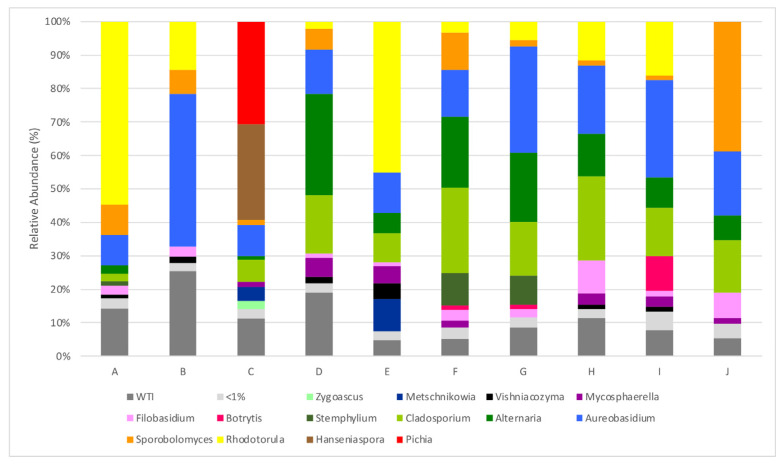
Relative abundances (%) of dominant sequences (>1%) assigned to genus level that were identified in samples based on partial sequence analysis of the Internal Transcribed Spacer 2 regions of the rRNA gene. WTI refers to the percentage of sequences without taxonomic attribution to the specified taxonomic level. A–J refers to the origin of the sequenced sample, as described in Table 1.

The sequencing of the metagenomic DNA extracted from the microbiota at the surface of *Alvarinho* grape berries from ten different locations across continental Portugal, made it possible to obtain a total of 442,191 ITS rRNA gene sequences, corresponding to 6 phyla, 189 families, 337 genera and 754 species. It is, however, important to bear in mind that an overestimation of the number of species may occur due to methodological limitations, as already discussed in Section 3.1. In fact, the accuracy and effectiveness of utilizing a universal gene to identify and classify fungal species is still a major obstacle in metabarcoding [29], with intraspecies barcode diversity and the existence of species with chimeric genomes as factors limiting the accuracy of the results [30]. At the genus level, an average of 102 different genera were identified for samples from parcels A to J, with a maximum of 142 genera in parcels from the Chaves and Vidigueira sub-regions and a minimum of 54 in parcel B from the Monção and Melgaço sub-region. Although there is a significant variability between sampled plots, some genera are particularly abundant and are present in most samples sequenced. For an abundance threshold of 5% of total reads, *Aureobasidium*, the ascomycetous dimorphic fungus, which is technologically irrelevant in enological terms, is the only genus present in all parcels, as can be observed in Figure 2. However, regarding the prevalence of certain types of yeast on the surface of grape berries, it is necessary to consider a significant diversity of results in the bibliography, depending on various factors such as temperature and other edapho-climatic factors, the stage of maturation, the variety of the grapes, the geographical location and pesticide applications. Prakitchaiwattana et al. [31] also found *Aureobasium* to be the most dominant genus present in the surface of the berries, followed by *Cryptococcus*, *Rhodotorula* and *Rhodosporidium*. However, the populations are variable with the grape variety. In climates with lower temperatures, Yanagida et al. [32] found that *Cryptococcus* and *Rhodotorula* genus were dominant in relation to ascomycetous yeasts.

*Cladosporium* is present in all sequenced samples; however, only eight of the ten characterized parcels showed an abundance above 5%. Similarly, *Alternaria* is also present in all characterized parcels; however, only seven of the ten parcels showed an abundance above 5% of the total reads. All of the above-mentioned genera are common members of the epiphytic flora found on the surface of grape berries, and some of them are related to grape damage, as is the case for the saprophytic mold *Cladosporium* [5] and *Alternaria*. Some species of the latter are responsible for damaging berries and bunch rot [33]. In a recent study of the epiphytic fungal community in *Vitis vinifera* of the Portuguese wine regions, *Alternaria* (31%) and *Cladosporium* (21%) were the most prevalent fungi present. *Aureobasidium* is also present in high amounts, although it is enologically irrelevant [34]. The presence of some plant pathogens, such as *Microstroma album*, *Bipolaris* spp., *Epicoccum nigrum*, *Fusarium* spp., *Sydowia polyspora*, *Phlebia* and *Tilletiopsis*, is observed although without any particular distribution among the parcels from the several regions and in very low amounts (mean of 0.02 ± 0.04% relative abundance of total reads). Of particular relevance is the presence of *Botrytis cinerea* in high amounts in parcel I from the Dão region (10.4% of total reads).

### 3.3. Influence of the Region/Sub-Region on the Microbial Composition

**Figure 3:**
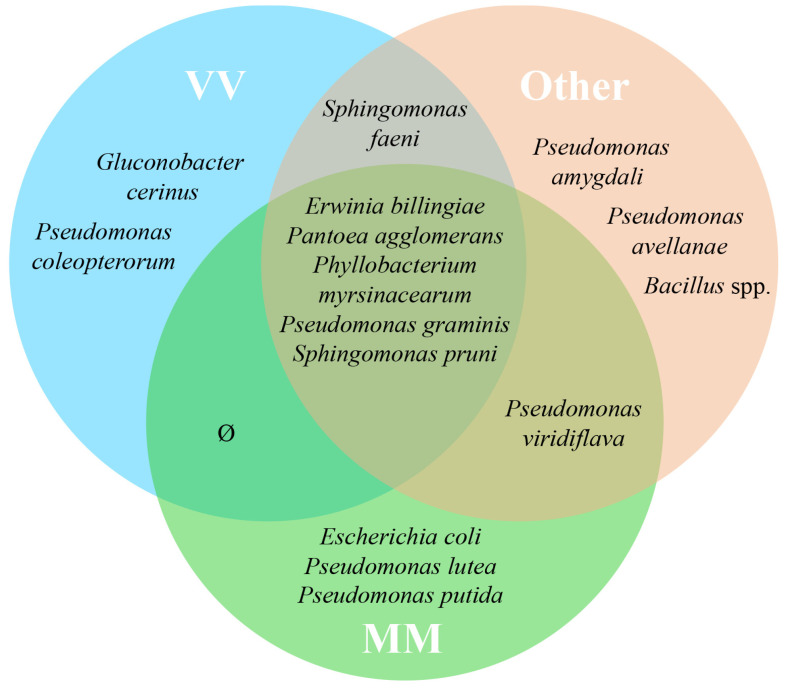
Venn diagram representing shared taxa of bacteria found on grape surface from ten Portuguese vineyard parcels from different regions/sub-regions, Monção and Melgaço (green area), Vinho Verde region (blue area) and Other (red area). Only taxa that are common to 100% of the parcels from a specific group (MM, VV or Other) are represented.

**Figure 4:**
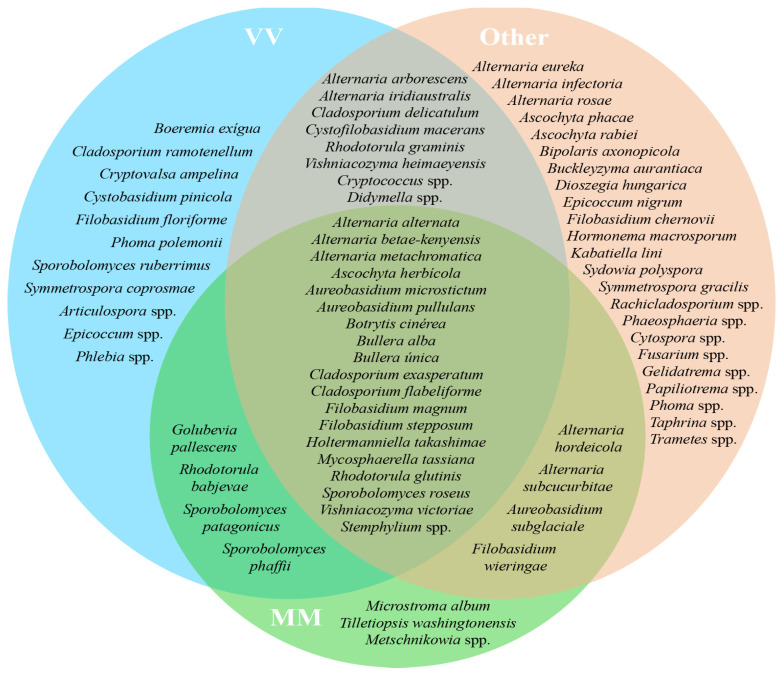
Venn diagram representing shared core taxa of fungi found on grape surface from ten Portuguese vineyard parcels from different regions/sub-regions, Monção and Melgaço (green area), Vinho Verde region (blue area) and Other (red area). Only taxa that are common to 100% of the parcels from a specific group (MM, VV or Other) are represented.

The origin of the grape variety *Alvarinho* is of the utmost relevance, not only economically but also for the identity of the sub-region of Monção and Melgaço. The wines produced with *Alvarinho* grapes are usually premium and known for their unique character. Of the approximately 3000 ha of *Alvarinho* variety planted in Portugal, 50% are in the sub-region of Monção and Melgaço [35,36]. Therefore, it was decided to create three different groups of plots—Group “MM”, which encompasses all the parcels sampled from the sub-region of Monção and Melgaço (parcels A, B, C and D); Group “VV” (parcels E, F and G), to which belong the other parcels from the VV region but not from Monção and Melgaço; Group “Other” (parcels H, I and J), with all the other parcels from the regions of Dão, Alentejo and Trás-os-Montes. Venn diagrams illustrate the shared taxa (at the genus level) of bacteria (Figure 3) and fungi (Figure 4) based on 100% of the samples taken in each group (MM, VV and Other) without considering their abundance. Even a taxon present in small amounts in all parcels in its group was considered. From this analysis, a group of five transversal taxa were found in all analyzed parcels, including *Erwinia*, *Pantoea*, *Phyllobacterium*, *Pseudomonas* and *Sphingomonas*, whereas *Escherichia coli* and two *Pseudomonas* spp. were common to all parcels only from the Monçao and Melgaço sub-region. Regarding fungi, a group of 17 species from 13 genera was found to be associated with all sampled parcels, whereas *Microstroma*, *Tilletiopsis* and *Metschnikowia* were common to all parcels only for the Monção and Melgaço sub-region. The ubiquitous presence of *Metschnikowia* in the surface of grapes from the Monção and Melgaço sub-region might have some enological relevance. In fact, earlier studies proved that some *Metschnikowia* species have a positive impact on the sensorial attributes of the wine produced in its presence. This impact is mainly due to the production of desirable volatile secondary metabolites [37,38,39,40], in particular during the initial phase of the fermentation when the ethanol content is still low enough to allow its metabolism. Ruiz et al. [39] have used a specific strain of *Metschnikowia pulcherrima* in a sequential inoculation of *Verdejo* must combined with *Saccharomyces cerevisiae*. They have obtained wines with a lower ethanol content but fruiter and fresher taste from the sensorial point of view; these wines present higher levels of 4-methyl-4-sulfanylpentan-2-one. Mixed fermentations with *Metschnikowia pulcherrima* and *Saccharomyces cerevisiae* have also been tested with several other grapevine varieties, including *Alvarinho*. Duarte and colleagues [40] performed sequential fermentations using *Metschnikowia pulcherrima* and fourteen grape varieties, red and white, and the produced wines were analyzed. The analyses showed that there was generally a reduction in alcohol content and an increase in glycerol, reducing sugars and total dry matter. The sensorial impact was significant in particular for three of the varieties tested. The potential relevance of the genus *Metschnikowia* in enology, despite its low to moderate fermentative power and low resistance to ethanol, has been recently reviewed [37]. The impact of *Metschnikowia* on the physicochemical parameters of the wine is vast (namely total acidity, volatile acidity, aroma compounds, polysaccharides and mannoproteins, anthocyanins, polyphenols and color). Therefore, it also has a vast impact on the sensorial attributes of the wines produced in its presence. 

**Figure 5:**
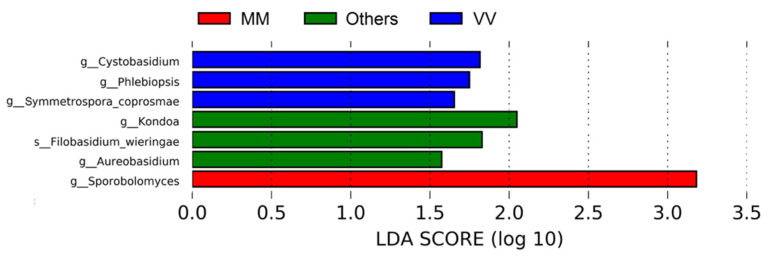
Linear Discriminant Analysis (LDA) scores computed for fungi features differentially abundant in parcels from MM, VV and Other groups.

A LEfSe analysis (Figure 5) on the data revealed seven divergent taxa between the three groups of parcels mentioned above. The most significant feature is related to the anamorphic basidiomycetous yeast *Sporobolomyces*, specifically in MM plots when compared to the rest, but without known enological relevance. This yeast is commonly present in many vineyards in different parts of the world [5,41].

**Figure 6:**
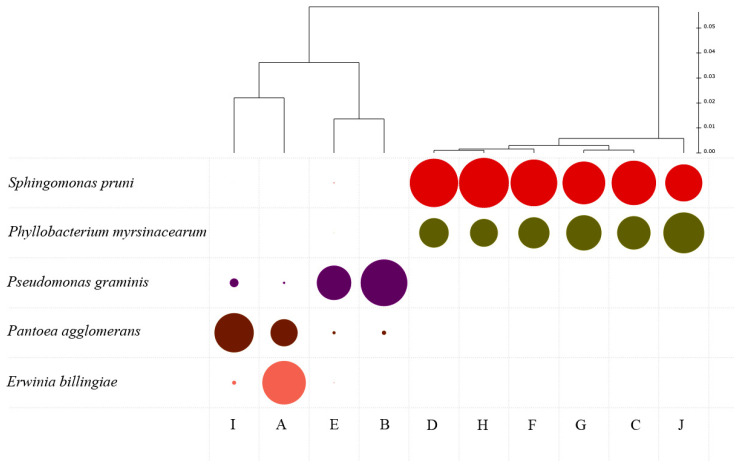
Clustering dendrogram considering the most dominant bacterial groups present in the different plots (A–J), and size of colored circles reflecting relative abundance of each taxon.

The dendrogram produced by a cluster analysis (Figure 6) using the core top dominant groups (>5% relative abundance) of bacteria present in the parcels clearly shows two different clusters in terms of relative abundance: one dominated by the *Sphingomonas pruni* group and the *Phillobacterium myrsinacearum* group and the other containing *Pseudomonas graminis*, *Pantoea agglomerans* and *Erwinia billingiae* groups. *Sphingomonas* species have been found to survive wine fermentation, but the effects of its presence are unknown [42]. Most of the above-mentioned genera have been identified and previously associated with grapevine. Despite a general description as plant pathogens, some strains have also been shown to have probable beneficial activity towards some relevant vineyard pathogens, such as *Botrytis cinerea*. *Phillobacterium* species are considered by some authors to be a plant probiotic, in particular for plants whose fruits are consumed raw. This probiotic effect may influence plant health and productivity [43], but its role in wine production is unknown. The Enterobacteriaceae *P. agglomerans* is commonly described as an epiphytic plant pathogen [44] which can cause disease in crops [45]. However, some strains have been found to act as a biocontrol agent of several plant pathogens [46,47]. The role of *Pseudomonas* spp. in the reduction of grapevine diseases through a mechanism of induction of systemic resistance has also been demonstrated [48].

As recently reviewed, microbial diversity is affected by grape variety and maturity, as well as many edaphoclimatic factors, geographical location and agronomic practices [49]. In the present study, all the berry samples belong to the same variety (*Alvarinho*) and were collected shortly before the harvesting. However, the ten plots where the sample collection occurred are dispersed geographically throughout the country (continental Portugal). A regional signature for the microbial consortium has been suggested by some authors, mainly because the determination of epiphytic flora has been performed via high throughput sequencing. Bokulich and colleagues [1] have demonstrated that microbial biogeography is nonrandomly associated with cultivars but also with regional factors. The study of microbial consortia associated with *Chardonnay* musts across California has revealed high positive and negative correlations of certain climatic factors such as net precipitation, relative humidity, maximum temperature and average low temperature with selected taxa, such as *Mycosphaerellaceae*, *B. fuckeliana*, *Pseuodomonadales*, *Moraxellaceae*, *Cladosporium*, *Penicillium*, *Pseudomonas*, *Enterobacteriaceae* and *Leuconostocaceae* (*Oenococcus oeni*). Vitulo and colleagues [50], however, have found that geographic location influences the grape microbiome but to a much lesser extent than the bark microbiome. The grape microbiome, in this case from *Dolcetto* and *Sangiovese* varieties in parcels from Piedmont and Tuscany in Italy, is much more influenced by anthropogenic factors such as agronomic management. The microbial consortium at the surface of *Cannonau* berries was found to be different according to the geographic location of parcels in Sardinia, which is also in Italy. In Catalonia, Spain, the bacterial diversity of *Grenache* and *Carignan* grape surface was found to be related to geographical situation and orientation of the parcels [51]. Castrillo et al. [52,53] have performed the isolation and identification of yeasts in grapes and musts from different varieties, including *Albariño*, in Galician vineyards, which is near Portugal, and have found the existence of biogeographic patterns of yeast populations.

Additionally, in our study, we could observe that the microbial communities associated with the surface of the berries are different between plots of the same region/sub-region as well between plots of different regions. Moreover, no relation between the microbial consortium and the geographic location or demarcated wine regions could be observed (apart from a lower fungi diversity in the Monção and Melgaço sub-region and the ubiquitous presence of *Metschnikowia* spp. in this region). However, an increase in the number of plots analyzed *per* demarcated region would be desirable for a future study. Another area of interest for the future would be the study of the putative relationship of the microbiome not only with the classification of the parcels in terms of the demarcated region to which they belong but also to edapho-climatic factors. It is also important to consider that the results obtained refer to the microbiome and not to the microbiota present on the surface of the grapes, because the high-throughput sequencing of specific DNA regions from metagenomic DNA will inevitably include DNA from live cells but also from dead cells. Furthermore, the presence of microbial DNA from environmental contaminants, dust and surrounding plants is likely to be present as well.

### 3.4. Alpha and Beta Diversity Analyses

**Figure 7:**
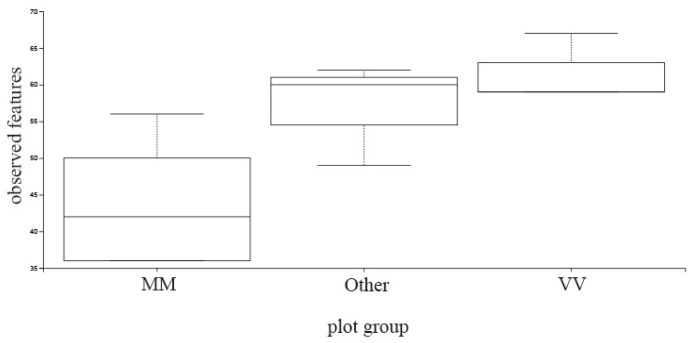
α-diversity box-plot considering OTU number in parcels from Monção and Melgaço region (MM), Vinho Verde region (VV) and all the rest parcels analyzed (Other).

The three groups previously formed (MM, VV and Other) were analyzed in terms of α and β-diversity. Different indices were calculated, and bacterial richness, evenness and phylogenetic diversity were examined (Supplementary Tables S1 and S2). Bacterial richness and diversity estimators did not show any specific pattern with statistical significance considering the groups formed. Differences seem to be more parcel-specific than region-specific. In terms of fungi diversity, MM parcels stood out in relation to the rest of VV plots in terms of the number of OTUs (*p* = 0.030754), as can be observed in Figure 7. Interestingly, the lower diversity of fungi in Monção and Melgaço parcels is coincident with the ubiquitous presence of *Metchnikowia* spp. in the same plots. This ascomycetous yeast, or at least some of its strains, is reported to display a strong biocontrol activity against several yeast and filamentous fungi, such as *Brettanomyces*, *Pichia*, *Penicillium*, *Aspergillus* and *Fusarium* species [37]. Two species found in the samples from Monção and Melgaço, *Metschnikowia chrysoperlae* [54] and *Metschnikowia pulcherrima* [55], are described as having biocontrol potential. Pulcherrimin, an iron-containing dipeptide compound which has the potential to inhibit the growth of other microorganisms, is produced by *M. pulcherrima* [55,56] and is also found on others species such as *M. andauensis* [57]. *M. pulcherrima* has been found to be an effective means of protecting grapes and other crops from post-harvest rot caused by *Botrytis cinerea* and other postharvest pathogens [58]. Its close relative, *M. fructicola*, is also capable of preventing postharvest diseases in grapes [59], as it is used as a biocontrol agent of postharvest diseases [60].

No ecological dissimilarity is observed between MM, VV and Other plots, as can be observed through the results obtained for β-diversity estimators such as Bray–Curtis (Supplementary Tables S3 and S4).

## 4. Conclusions

The number of published studies concerning the microbial characterization associated with the grapes of the *Alvarinho* variety is scarce, and, as far as the authors are aware, the manuscript presents the most extensive study to date regarding the identification of the microbial population associated with this variety and the first metagenomic approach. Although this study has contributed to a deeper understanding of the main microbial flora (and its diversity) associated with the surface of *Alvarinho* grapes in different geographically dispersed regions in Portugal, some limitations can be pointed out, namely the fact that the study was restricted to a single season and the number of plots per region is relatively low. The repetition of the study with a greater number of plots per region during another season integrated with edapho-climatic data and not only geographic dispersion could thus be relevant. The data presented do not allow for conclusions regarding a relationship between taxonomic composition and geographic distance and/or the sampled, demarcated regions, as it was observed that variance between parcels within the same region is somehow similar to the one observed between regions. However, at least two features may distinguish the main *Alvarinho* region in Portugal, Monção and Melgaço. These features are the ubiquitous presence of *Metschnikowia* spp. in all parcels and the phylogenetic diversity of fungi. However, the relevance of those features to the *terroir* that is recognized for the production of the light, acidic, refreshing, high-quality, monovarietal *Alvarinho* wines in the region of Monção and Melgaço is yet to be demonstrated.

## Figures and Tables

**Table 1:** Origin of the samples. Parcel designation and the identification of its corresponding regions/sub-regions.

Parcel	Region	Sub-Region
A, B, C, D	Vinho Verde	Monção and Melgaço
E	Vinho Verde	Lima
F	Vinho Verde	Cávado
G	Vinho Verde	Basto
H	Trás-os-Montes	Chaves
I	Dão	Terras de Azurara
J	Alentejo	Vidigueira

## Data Availability

Raw reads were deposited in SRA database under BioProject PRJNA885087.

## Supplementary Materials

The following supporting information can be downloaded at: https://www.mdpi.com/article/10.3390/biology12020146/s1, Figure S1: Regions (and sub-regions) sampled; Table S1: Summary of α-diversity significance data (bacteria); Table S2: Summary of α-diversity significance data (fungi); Table S3: Summary of β-diversity significance data (bacteria); Table S4: Summary of β-diversity significance data (fungi).
