# Supplementary material for: Epiphitic Microbiome of Alvarinho Wine Grapes from Different Geographic Regions in Portugal
Source: Biology (Basel). 2023 Jan 18;12(2):146. doi: 10.3390/biology12020146 (PMC9952175; doi:10.3390/biology12020146)
Supplement: Supplementary file 1 [file biology-12-00146-s001.zip › biology-2106288-supplementary.pdf]

Supplementary material

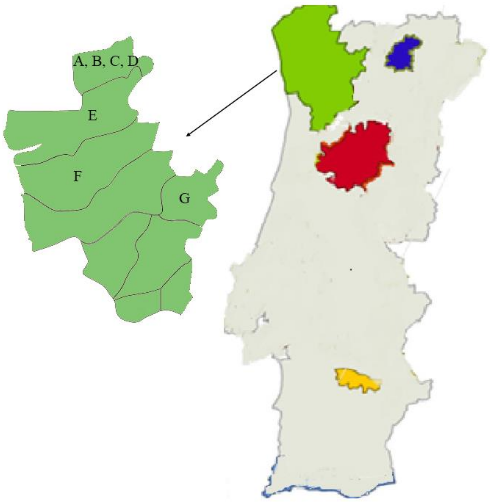

Figure S1. Regions (and sub-regions) sampled. Green: Vinho Verde region (Monção & Melgaço, Lima, Cávado, Basto); Blue: Trás-Os-Montes region (Chaves); Red: Dão region Terras de Azurara); Yellow: Alentejo region (Vidigueira). On the left, an ampliation of the Vinho Verde regions with corresponding delimited sub-regions and the location of sampled parcels in this region.

Table S1. Summary of -diversity significance data (bacteria).

		Evenness		Faith		Observed OTUs		Shannon	
		p-value	q-value	p-value	q-value	p-value	q-value	p-value	q-value
Group	Overall	0.283912	–	0.704688	–	0.688853	–	0.815017	–
	MM vs VV	0.288844	0.433267	0.723674	0.723674	0.479500	0.719250	0.723674	0.827259
	MM vs Other	0.157299	0.433267	0.479500	0.723674	0.479500	0.719250	0.479500	0.827259
	VV vs Other	0.512691	0.512691	0.512691	0.723674	0.827259	0.827259	0.827259	0.827259

Table S2. Summary of -diversity significance data (fungi)

		Evenness		Observed OTUs		Shannon	
		p-value	q-value	p-value	q-value	p-value	q-value
Group	Overall	0.785913	–	0.059573	–	0.688853	–
	MM vs VV	0.479500	0.827259	0.030754	0.092261	0.479500	0.719250
	MM vs Other	0.723674	0.827259	0.074462	0.111693	0.479500	0.719250
	VV vs Other	0.827259	0.827259	0.824778	0.824778	0.827259	0.827259

Table S3. Summary of -diversity significance data (bacteria)

		Bray-Curtis		Jaccard		Weighted-Unifrac		Unweighted-Unifrac	
		p-value	q-value	p-value	q-value	p-value	q-value	p-value	q-value
Group	Overall	0.939	–	0.882	–	0.905	–	0.929	–
	MM vs VV	0.682	1.0	0.569	0.908	0.800	0.943	0.978	0.978
	MM vs Other	1.0	1.0	0.908	0.908	0.943	0.943	0.722	0.978
	VV vs Other	0.911	1.0	0.702	0.908	0.693	0.943	0.804	0.978

Table S4. Summary of -diversity significance data (fungi)

		Bray-Curtis		Jaccard	
		**p-value**	**q-value**	**p-value**	**q-value**
Group	Overall	0.502	–	0.608	–
	MM vs VV	0.707	0.913	0.673	0.796
	MM vs Other	0.213	0.639	0.523	0.796
	VV vs Other	0.913	0.913	0.796	0.796
